# Associated factors with functional disability and health-related quality of life in Chinese patients with gout: a case-control study

**DOI:** 10.1186/s12891-017-1787-7

**Published:** 2017-11-03

**Authors:** Ting Fu, Haixia Cao, Rulan Yin, Lijuan Zhang, Qiuxiang Zhang, Liren Li, Zhifeng Gu

**Affiliations:** 1 Research Center of Clinical Medicine, Affiliated Hospital of Nantong University, Nantong, Jiangsu People’s Republic of China; 2 Department of Rheumatology, Affiliated Hospital of Nantong University, Nantong, Jiangsu People’s Republic of China; 3 School of Nursing, Nantong University, Nantong, Jiangsu People’s Republic of China

**Keywords:** Gout, Functional disability, Health-related quality of life, Depression, Anxiety

## Abstract

**Background:**

Gout is a painful, inflammatory disease that may cause decreased function and health-related quality of life (HRQoL). Limited study did not take the influence of gout characteristics and anxiety on HRQoL into consideration and there are no studies associated with functional disability in individuals with gout from China. This study aims to investigate the related factors of functional disability and HRQoL in gout patients recruited from China.

**Methods:**

A total of 226 consecutive gout patients and 232 age- and gender-matched healthy individuals were involved in the study. A series of questionnaires (the Short Form 36 health survey, the Patient Health Questionnaire, the Generalized Anxiety Disorder questionnaire, the 10 cm Visual Analog Scale, and the Health Assessment Questionnaire-Disability Index) were applied. Blood samples were taken to examine the level of serum uric acid. Independent samples *t*-tests, Chi square tests, U test, Spearman rank correlation, logistic regression modeling, and linear regression were used to analyze the data.

**Results:**

After adjusted demographic variables, individuals with gout have poorer HRQoL compared to healthy controls. Univariate tests presented that patients with functional disability had longer disease duration, more frequent flares/last year, more severe total pain, more number of tophi, higher degree of depression and anxiety, with a trend toward diabetes, the treatment of colchicine and corticosteroids use, compared to patients without functional disability. Meanwhile, place of residence, hypertension, DM, disease duration, cardiovascular disease, number of flares/last year, total pain, more number of tophi, presence of tender joints, depression, anxiety, currently using colchicine and corticosteroids were correlated significantly with HRQoL. Additionally, multiple regression analysis identified severe pain, depression, and colchicine use as predictors of functional disability. Cardiovascular disease, total pain, number of flares/last year, presence of tender joints, depression, anxiety, colchicine and corticosteroids use contributed to low HRQoL.

**Conclusions:**

After adjusted demographic variables, gout subjects have poorer HRQoL compared to healthy controls. Chinese gout population experiencing poor HRQoL and functional disability were likely to suffer from gout-related features and psychological problems. The results underscore the need of effective interventions including psychological nursing and appropriate treatment approaches to reduce their functional disability and improve their HRQoL.

## Background

Gout is a type of inflammatory arthritis that is triggered by the crystallization of uric acid within the synovial fluid of joints and other tissues [1]. The prevalence of gout is increasing and currently is commonest inflammatory arthritis in men [2, 3]. Gout individuals regularly suffer from signs and symptoms of gout (e.g., severe pain, frequent flares, more number of tophi, more munber of involved joints), which results in the impairments of function and health-related quality of life (HRQoL) [4–7]. Previous studies have revealed that gout individuals had impaired overall HRQoL compared with general population [8, 9]. It has been reported that socioeconomic status and disease-related characteristics may have an impact on functional disability and HRQoL in subjects with gout [8, 10, 11]. Current evidence estimated that gout patients affected by severe pain, tophi, polyarticular disease, a great number of attacks, and more number of involved joints frequently had functional disability and worse HRQoL [11–13]. Additionally, recent findings identified that patients with gout experienced a higher risk of psychological problems [5, 14]. Depression and/or anxiety are known to be independent risk factors for functional disability and HRQoL among patients with other chronic diseases [15, 16]. However, it remains unclear whether psychological status has an effect on HRQoL. Only the report of Chandratre et al. found that worse HRQoL was seen in gout patients with psychological disorders and depression was associated with functional disability [17]. Thus, identifying factors, especially psychological status, related to functional disability and HRQoL in gout patients is of great importance.

A meta-analysis indicated that gout is experienced by 1.14% adults in China [18]. At present, the majority researches related HRQoL of gout from China were intervention studies. The only paper to have determined the related factors of HRQoL was conducted among a Chinese sample, without taking into consideration the influence of gout characteristics and anxiety [19]. Importantly, it has been reported that gout commonly contributes to tophi and joint impairment impacting functional disability [13]. Unfortunately, no studies associated with functional disability of Chinese gout patients have been attempted.

Given the above-mentioned considerations, the objectives of this study were (1) to evaluate the functional disability and HRQoL in Chinese gout patients; (2) to investigate the effects of demographic variables, disease parameters, and psychological status on functional disability and HRQoL in gout population recruited from China.

## Methods

### Participants

Patients living with gout were outpatients or inpatients from the Affiliated Hospital of Nantong University from November 2015 to January 2017. The consecutive patients with gout all fulfilled the 1977 American College of Rheumatology preliminary criteria for the diagnostic of gout [20]. The exclusion criteria for gout individuals: (1) age ≤ 18 years old; (2) cannot finished the questionnaires. Out of the 229 patients approached, three patients did not finish the questionnaires. Finally, 226 patients were included in this case-control study. Response rate of effective questionnaires was 98.69%.

The controls were selected from family numbers of patients or from a population attending for an annual examination in the Affiliated Hospital of Nantong University. They were matched to the gout patients by age and gender by the way of frequency matching. Age- and gender-matched healthy individuals were included if they did not living with systemic diseases or psychiatric disorders.

This case-control study was approved by the Ethics Committee of the Affiliated Hospital of Nantong University. Written informed consents were obtained from all of the participants, according to the Declaration of Helsinki.

### Demographic and clinical variables

Demographic parameters involved the following: gender, age, body mass index (BMI) (kg/m^2^), waist-hip ratio (WHR), place of residence, marital status, education, employment status, and income/year, and medical insurance coverage.

Clinical parameters contained comorbidities (defined by self-reported or physician diagnosis), disease duration, number of flares/last year, and medications by viewing medical records or asking patients. Stage of gout (including asymptomatic hyperuricemia, acute phase, inter-critical stage, and chronic phase), number of tophi, presence of painful joints and swollen joints were obtained by the same clinician for all patients. Blood samples were taken to examine the level of serum uric acid (sUA) at the succeeding visit.

### Self-reported questionnaires

The Visual Analogue Scale (VAS) was used to assess total pain. A higher score indicating more severe pain [12].

Functional disability was evaluated by the Health Assessment Questionnaire -Disability Index (HAQ-DI) [21, 22]. Previous studies have confirmed that the HAQ-DI was a reliable and valid instrument for studies measuring disability for several forms of arthritis including gout [23, 24]. Disability was considered to be present for participants with a HAQ-DI score ≥ 0.5 [13, 25].

The Chinese version of Generalized Anxiety Disorder (GAD-7) questionnaire and Patient Health Questionnaire (PHQ-9) were used to measured levels of anxiety and depression [26, 27]. The GAD-7 and PHQ-9 have both been shown to be capable of screening for their respective conditions and are valid measures of clinically diagnosed anxiety and depression [28]. Scores of <10 represent “no anxiety” or “no depression” and scores of ≥10 represent the presence of anxiety or depression for each measure [28, 29].

The Short Form 36 health survey (SF-36) was used to measure HRQoL in the past 4 weeks. It assessed eight domains: physical function; role limitations due to physical problems; body pain; general health perception; energy/vitality; social function; role limitations due to emotional problems; mental health. Z-transformed and normalized domain scores were grouped into Physical Component Summary (PCS) and Mental Component Summary (MCS) [30]. Li et al. suggested that the Chinese version of the SF-36 functioned in the general population by scaling construction and scoring assumptions, validation, and normalization [31].

### Data collection

Resultant Chinese paper-based questionnaires were administered to participants under physician supervision in a single sitting, lasting from 15 to 20 min. Results were added to a computer database by two research assistants and double checked against the original data prior to analysis.

### Statistical analysis

Descriptive measures are presented as means and standard deviation (SD), frequencies (%), or median and interquartile range (IQR) based on their type and distribution. The differences in terms of continuous and categorical variables that are studied in gout patients who are grouped as individuals with functional disability and without functional disability were evaluated with the two-tailed *t* test, chi-square test and/or Mann-Whitney U test, as well as the differences of demographic variables between the gout patients and the controls. After adjusted demographic variables by multiple logistic regression, the differences of HRQoL and psychological status between gout group and controls were analyzed. The association of demographic variables, disease parameters, psychological status, and HRQoL were examined with Spearman rank correlation analysis. Variables shown to be significant in the univariate tests were included into multiple regression with functional disability and HRQoL as the dependent variables, respectively. Statistical significance was considered when *p* < 0.05 (two-sided). All analyses were performed using SPSS version 20.0.

## Results

### Participant characteristics

As three gout patients and four healthy controls did not finish the questionnaires, 226 gout cases and 232 age- and gender-matched healthy individuals were involved in the current study. Table 1 presented the baseline participant characteristics in our research. Of the 226 gout patients, the mean (SD) age was 53.18(15.77) years and 94.7% were male. Significant difference were found in the BMI, WHR, place of residence, income/year, and medical insurance between the gout subjects and health controls (*p* < 0.05). According to the cut-off scores, depression disorder was presented in 15.0%, 5.3% had anxiety, and 21.2% reported functional disability. After adjusted demographic variables, patients with gout had significantly higher risk of depressive disorder and lower HRQoL, when compared with control subjects (*p* < 0.05). The two groups did not differ in terms of the prevalence of anxiety (*p* > 0.05). The gout characteristics were summarized in Table 2. Amongst the gout participants, median VAS score was 4.25(0–7.75) and median number of flares/last year was 3(1.25–12). A lot of subjects had other comorbidities, included hypertension (42.5%), DM (10.2%), and cardiovascular disease (9.7%). More than half of patients were currently taking colchicine and 31.4% were been treated with corticosteroids.

**Table 1:** Baseline characteristics in gout patients and healthy controls

	Gout (*n* = 226)	Controls (*n* = 232)	*p*
Gender, male^b^	214 (94.7)	218 (94.0)	0.738
Age (years)^a^	53.18 ± 15.77	51.21 ± 13.48	0.153
BMI (kg/m^2^)^a^	25.40(23.28–27.74)	23.88(22.45–25.95)	*<0.001*
WHR^C^	0.95(0.91–0.98)	0.94(0.89–0.96)	*<0.001*
Place of residence^b^			*<0.001*
City	127 (56.2)	87 (37.5)	
Country	99 (43.8)	145 (62.5)	
Marital status^b^			0.290
Married	209 (92.5)	208 (89.7)	
Single	17 (7.5)	24 (10.3)	
Education^b^			0.112
≤ 9 years	104 (46.0)	124 (53.4)	
>9 years	122 (54.0)	108 (46.6)	
Employment status^b^			0.532
Employed	177 (78.3)	176 (75.9)	
Unemployed	49 (21.7)	56 (24.1)	
Income/year (RMB)^b^			*<0.001*
<15,000	83 (36.7)	61 (26.3)	
15,000–33,000	75 (33.2)	57 (24.6)	
>33,000	68 (30.1)	114 (49.1)	
Medical insurance, yes^b^	216 (95.6)	170 (73.3)	*<0.001*
Disability (HAQ-DI ≥ 0.5 score)^b^	48 (21.2)		
Depression (PHQ-9 ≥ 10 score)^b^	34 (15.0)	14 (6.0)	*0.001*
Anxiety (GAD-7 ≥ 10 score)^b^	12 (5.3)	10 (4.3)	0.358
SF-36 components score			
PCS^C^	43.88 (31.50–62.75)	88 .10 (80.88–93.00)	*<0.001*
MCS^C^	62.23 (48.27–83.31)	83.44 (74.31–88.50)	*<0.001*

SD: standard deviation; IQR: interquartile range; BMI: body mass index; WHR: waist-hip ratio; VAS: visual analog scale; HAQ-DI: health assessment questionnaire-disability index; sUA: serum uric acid; PHQ-9: patient health questionnaire; GAD-7: generalized anxiety disorder; SF-36: the Short Form 36 Health Survey; PCS: physical components summary; MCS: mental components summary

*p* values were obtained with the two-tailed *t* test, chi-square test and/or Mann-Whitney U test based on the type and distribution of variables

*P* numbers of entries with significance are shown in italics

^a^Mean ± SD. ^b^Number (percentage) .^C^Median (IQR)

**Table 2:** Disease-related characteristics in gout patients

	Gout (*n* = 226)	
Comorbidities^b^		
Hypertension	96 (42.5)	
DM	23 (10.2)	
Hyperlipidemia	25 (11.1)	
Cardiovascular disease	22 (9.7)	
Nephropathy	17 (7.5)	
Disease duration (year)^C^	5 (2–12)	
Number of flares/last year^C^	3 (1.25–12)	
Stage of gout^b^		
Asymptomatic (hyperuricemia)	8 (3.5)	
Acute phase	128 (56.6)	
Intercritical phase	37 (16.4)	
Chronic phase	53 (23.5)	
Number of tophi^b^		
No	136 (60.2)	
1	37 (16.4)	
≥ 2	53 (23.5)	
sUA (μmol/L)^a^	468.15 ± 116.59	
Presence of tender joints, yes^b^	190 (84.1)	
Presence of swollen joints, yes^b^	162 (71.7)	
Total pain (VAS)^C^	4.25(0–7.75)	
Urate lowering therapy^b^		
Allopurinol	16 (7.1)	
Febuxostat	80 (35.4)	
Any anti-inflammatory drugs^b^		
Colchicine	146 (64.6)	
NSAID	105 (46.5)	
Corticosteroids	71 (31.4)	

SD: standard deviation; IQR: interquartile range; DM: diabetes; NSAID: non-steroidal anti-inflammatory drugs

^a^Mean ± SD^b^Number (percentage)^C^Median (IQR)

### Comparison of gout patients with and without functional disability

Table 3 described the differences of demographic, clinical, and psychological variables in gout cases with and without functional disability after researchers adjusted demographic variables. The proportion of DM, colchicine and corticosteroids use in the group with functional disability was significantly higher than the group without functional disability (*p* < 0.05). In addition, subjects with functional disability were likely to suffer from long disease duration, frequent flares/last year, severe total pain, more number of tophi, depressive and anxious symptoms (*p* < 0.05).

**Table 3:** Comparison of gout patients with and without disability

	HAQ-DI < 0.5 score (*n* = 178)	HAQ-DI ≥ 0.5 score (*n* = 48)	*p*
Gender, male^b^	168 (94.4)	46 (95.8)	0.691
Age (years)^a^	52.83 ± 15.64	54.46 ± 16.34	0.527
BMI (kg/m^2^)^a^	25.68 ± 4.17	25.51 ± 4.28	0.807
WHR^C^	0.95(0.91–0.98)	0.96(0.94–0.98)	0.375
Place of residence^b^			0.518
City	102 (57.3)	25 (52.1)	
Country	76 (42.7)	23 (47.9)	
Marital status^b^			0.392
Married	166 (93.3)	43 (89.6)	
Single	12 (6.7)	5 (10.4)	
Education^b^			0.109
≤ 9 years	77 (43.3)	27 (56.2)	
>9 years	101 (56.7)	21 (43.8)	
Employment status^b^			0.872
Employed	139 (78.1)	38 (79.2)	
Unemployed	39 (21.9)	10 (20.8)	
Income/year (RMB)^b^			0.153
<15,000	63 (35.4)	20 (41.7)	
15,000–33,000	56 (33.2)	19 (39.6)	
>33,000	59 (33.1)	9 (18.8)	
Medical insurance, yes^b^	168 (94.4)	48 (100.0)	0.093
Comorbilities^b^			
Hypertension	73 (41.0)	23 (47.9)	0.390
DM	14 (7.9)	9 (18.8)	*0.027*
Hyperlipidemia	21 (11.8)	4 (8.3)	0.497
Cardiovascular disease	15 (8.4)	7 (14.6)	0.269
Nephropathy	12 (6.7)	5 (10.4)	0.202
Disease duration (year)^C^	5.00(1.54–10.00)	7.00(3.00–14.79)	*0.020*
Number of flares/last year^C^	3.00(1.00–10.00)	8.00(2.88–24.25)	*0.003*
Stage of gout^b^			0.087
Asymptomatic (hyperuricemia)	7 (3.9)	1 (2.1)	
Acute phase	102 (57.3)	26 (54.2)	
Intercritical phase	33 (18.5)	4 (8.3)	
Chronic phase	36 (20.2)	17 (35.4)	
Total pain (VAS)^C^	3.00(0.00–7.00)	5.50(5.00–8.00)	*<0.001*
Number of tophi^b^			*0.027*
No	115 (64.6)	21 (43.8)	
1	27 (15.2)	10 (20.8)	
≥ 2	36 (20.2)	17 (35.4)	
sUA (μmol/L)^a^	462.79 ± 1165.80	488.02 ± 118.57	0.184
Presence of tender joints, yes^b^	146 (82.0)	44 (91.7)	0.105
Presence of swollen joints, yes^b^	125 (70.2)	48 (100.0)	0.349
Urate lowering therapy^b^			
Allopurinol	15 (8.4)	1 (2.1)	0.128
Febuxostat	59 (33.1)	21 (43.8)	0.173
Any anti-inflammatory drugs^b^			
Colchicine	108 (60.7)	38 (79.2)	*0.017*
NSAID	81 (45.5)	24 (50.0)	0.580
Corticosteroids	50 (28.1)	21 (43.8)	*0.038*
Depression (PHQ-9 ≥ 10 score)^b^	13 (7.3)	21 (43.8)	*<0.001*
Anxiety (GAD-7 ≥ 10 score)^b^	5 (2.8)	7 (14.6)	*0.004*

SD: standard deviation; IQR: interquartile range; BMI: body mass index; WHR: waist-hip ratio; DM: diabetes; VAS: visual analog scale; sUA: serum uric acid; NSAID: non-steroidal anti-inflammatory drugs, HAQ-DI: health assessment questionnaire-disability index; PHQ-9: patient health questionnaire; GAD-7: generalized anxiety disorder

*p* values were obtained with the two-tailed *t* test, chi-square test and/or Mann-Whitney U test based on the type and distribution of variables

*P* numbers of entries with significance are shown in italics

^a^Mean ± SD.^b^Number (percentage). ^C^Median (IQR)

### Correlations between demographic variables, disease parameters, psychological status, and HRQoL

As shown in Table 4, Spearman rank correlation coefficients were computed to identify the relationships among two dimensions of SF-36, demographic, clinical, and psychogenic variables in present sample of gout patients. Patients with cardiovascular disease, more number of flares/last year, severe total pain, more number of tophi, presence of tender joints, depression, anxiety, currently using colchicine and corticosteroids were more likely to have lower scores of PCS and MCS (*p* < 0.05). Additionally, place of residence, hypertension, DM, and disease duration were significantly related to PCS (*p* < 0.05).

**Table 4:** Correlations among demographic, clinical, psychological variables and SF-36 in gout patients

	PCS		MCS	
	r	*p*	R	*p*
Gender, male	0.036	0.587	0.044	0.509
Age (years)	−0.051	0.441	0.083	0.214
BMI (kg/m^2^)	0.077	0.247	0.063	0.344
WHR	−0.094	0.157	−0.044	0.515
Place of residence	−0.160	*0.016*	−0.110	0.100
Marital status	−0.037	0.578	−0.018	0.788
Education	0.099	0.140	0.065	0.331
Employment status	−0.008	0.907	0.035	0.599
Income/year	0.113	0.090	0.068	0.311
Medical insurance, yes	0.009	0.894	−0.043	0.525
Comorbidities				
Hypertension	−0.138	*0.038*	−0.033	0.621
DM	−0.140	*0.036*	−0.079	0.238
Hyperlipidemia	−0.005	0.941	0.031	0.643
Cardiovascular disease	−0.194	*0.003*	−0.182	*0.006*
Nephropathy	−0.018	0.785	−0.012	0.855
Disease duration (year)	−0.198	*0.003*	−0.066	0.324
Number of flares/last year	−0.292	*<0.001*	−0.174	*0.009*
Stage of gout	−0.066	0.322	−0.060	0.367
Total pain (VAS)	−0.349	*<0.001*	−0.251	*<0.001*
Number of tophi	−0.233	*<0.001*	−0.159	*0.017*
UA (μmol/L)	−0.083	0.216	−0.100	0.133
Presence of tender joints, yes	−0.175	*0.008*	−0.243	*<0.001*
Presence of swollen joints, yes	−0.108	0.105	−0.130	0.051
Urate lowering therapy				
Allopurinol	0.087	0.192	0.107	0.107
Febuxostat	0.049	0.467	0.005	0.942
Any anti-inflammatory drugs				
Colchicine	−0.157	*0.018*	−0.159	*0.017*
NSAID	−0.006	0.925	−0.025	0.712
Corticosteroids	−0.156	*0.019*	−0.228	*0.001*
Depression (PHQ-9 ≥ 10 score)	−0.366	*<0.001*	−0.407	*<0.001*
Anxiety (GAD-7 ≥ 10 score)	−0.195	*<0.001*	−0.290	*<0.001*

SD: standard deviation; IQR: interquartile range; BMI: body mass index; WHR: waist-hip ratio; DM: diabetes; VAS: visual analog scale; sUA: serum uric acid; NSAID: non-steroidal anti-inflammatory drugs, HAQ-DI: health assessment questionnaire-disability index; PHQ-9: patient health questionnaire; GAD-7: generalized anxiety disorder

*p* values were obtained with Spearman rank correlation analysisanalysis

*P* numbers of entries with significance are shown in italics

### Predictors of functional disability in gout patients

Stepwise multiple logistic regression analysis was conducted to investigate contributors of functional disability, as indicated in Table 5. Total pain, depression, and colchicine use were significantly accounted for functional disability.

**Table 5:** Stepwise multiple logistic regression analysis of HAQ-DI in gout patients

	β	SE	*p*	Exp(B)	Exp(B) 95% CI	
					Lower	Upper
Total pain (VAS)	0.133	0.054	*0.013*	1.142	1.028	1.268
Depression (PHQ-9 ≥ 10 score)	2.114	0.430	*<0.001*	8.280	3.565	19.233
Colchicine	0.915	0.431	*0.034*	2.496	1.073	5.804

HAQ-DI: health assessment questionnaire-disability index; PHQ-9: patient health questionnaire

*p* values were obtained with Stepwise multiple logistic regression analysisanalysis

*P* numbers of entries with significance are shown in italics

### Determinants of HRQoL in gout patients

As indicated in Table 6, the results of stepwise multiple linear regression analysis revealed that lower PCS score was predicted by depression, total pain, cardiovascular disease, number of flares/last year, and colchicine use. Depression, presence of tender joints, total pain, anxiety and corticosteroids use appeared to be indicators of lower MCS score. Finally, these variables explained 24.7% and 26.7% of the variance in PCS and MCS, respectively.

**Table 6:** Stepwise multiple linear regression analysis of SF-36 domains in gout patients

	β	SE	*t*	*P*	B (95% CI)		*F*	Adj R^2^
					Lower	Upper		
PCS							15.779	0.247
Depression (PHQ-9 ≥ 10 score)	−16.649	3.693	−4.508	*<0.001*	−23.928	−9.370		
Total pain (VAS)	−1.551	.372	−4.165	*<0.001*	−2.285	−0.817		
Cardiovascular disease	−12.562	4.342	−2.894	*0.004*	−21.119	−4.006		
Number of flares/last year	−0.129	0.058	−2.226	*0.027*	−0.244	−0.015		
Colchicine	−5.772	2.666	−2.165	*0.031*	−11.026	−0.518		
MCS							14.686	0.267
Depression (PHQ-9 ≥ 10 score)	−14.268	3.967	−3.596	*<0.001*	−22.088	−6.449		
Presence of tender joints, yes	−8.333	3.388	−2.460	*0.015*	−15.010	−1.657		
Corticosteroids	−7.758	2.581	−3.006	*0.003*	−12.844	−2.671		
Total pain (VAS)	−0.848	0.353	−2.404	*0.017*	−1.544	−0.153		
Anxiety (GAD-7 ≥ 10 score)	−13.452	6.102	−2.204	*0.029*	−25.477	−1.426		

VAS: visual analog scale; PHQ-9: patient health questionnaire; GAD-7: generalized anxiety disorder; SF-36: the Short Form 36 Health Survey; PCS: physical components summary; MCS: mental components summary

*p* values were obtained with stepwise multiple linear regression analysisanalysis

*P* numbers of entries with significance are shown in italics

## Discussion

Previous study has confirmed that patients with gout experienced poorer HRQoL compared with the norm, in both the emotional and physical health [12]. In agreement with the results of prior study, gout patients in the current study had poorer HRQoL than healthy controls [32]. More than a fifth of those gout cases had functional disability. Disease characteristics and psychological status significantly correlated with functional disability and HRQoL in Chinese gout population. Among the assessed parameters, depression, total pain, and colchicine use were both independent risk factors of functional disability of gout patients. In addition, cardiovascular disease, number of flares/last year, presence of tender joints, anxiety, corticosteroids and colchicine use were the significant causes of impaired HRQoL in gout patients. To our best knowledge, this was the first study to investigate the relationships among demographic, clinical, and psychological parameters and functional disability in Chinese patients with gout. Also, it was the first study to evaluate the effect of disease features and anxiety on HRQoL in Chinese gout population.

Existing studies have documented that demographic and clinical variables have an impact on functional disability and HRQoL in individuals with gout [10, 11, 33]. Similar to the results of Chandratre et al., the data in the present study showed that DM has a substantial impact on HAQ-DI. [17]. Grehh et al. identified that DM is associated with an increased incidence of functional disability, which is likely to further erode health status [34]. A population-based study analyzing the association between the HRQoL and rural residence reported that worse physical health among persons living in rural areas compared to those from urban settings [35]. In our study, place of residence was found to be associated with PCS domain of SF-36. Lahana et al. suggested that persons living in urban areas may have more access to health care services, material resources, physical and social environments, which help them enjoy longer and healthier lives [36]. Consistent with the reports of Roddy et al., there were significant relationships between comorbidities and HRQoL [37]. Patients with hypertension, DM, and cardiovascular disease have poorer HRQoL, which was similar with the results of other study [12]. Interestingly, multiple regression analysis showed that cardiovascular disease was a major contributor to PCS scale. Hsu et al. considered that cardiovascular disease can influence HRQoL through the mediation of anxiety and depression [38]. These findings suggested that it is necessary to best manage these commorbidities and pay attention to psychological health of gout patients.

There is evidence that gout characteristics (e.g., pain, number of flares, number of tophi, presence of painful joints) were associated with functional disability and HRQoL [9, 11, 12]. Our study also showed that number of flares/last year, disease duration, total pain and number of tophi were correlated with functional disability and HRQoL. Frequency flares/last year, more number of tophi, and presence of painful joints were associated with low PCS and MCS scores. Prior et al. suggested patients experiencing more frequent flares or attacks in a greater number of involved joints are much more likely to suffer from depressive symptoms, which may have an impact on their mental health [14]. Pain, tophi, and presence of painful joints and swollen joints may lead to limitation of activities. One study indicated that gout-specific variables significantly impacted on functional disability and HRQoL [4]. Another study has shown that gout patients with more number of flares were prone to experience greater activity impairment and both flares and tophi were associated with HRQoL [11]. Importantly, multiple regression analysis indicated that total pain and colchicine use were major contributors to functional disability. Presence of tender joints, total pain, and corticosteroids use explained higher HAQ-DI score. It is also possible that gout characteristics were associated with impairment in daily activities and higher costs, which have a dramatic influence on patients’ physical and mental health [39, 40]. It must be noted that variables (sUA, number of flares/last year, urate lowering therapy use) indicated undertreated gout, which maybe probably a strong driver of reduced HRQoL. Patients with gout in this cohort were recruited from the clinic, rather than from the community. Number of participants having well treated gout, without current flare was limited in this regard, which may explain our results. The findings suggested the importance of suitable interventions to help gout patients release the symptoms. Rome et al. have found a footwear intervention can reduce the level of foot pain and musculoskeletal disability in individuals with gout [41]. Furthermore, our study reported a negative correlation between disease duration and PCS in gout patients, which was consistent with previous study [4]. At last, patients currently treated with colchicines and corticosteroids were prone to have functional disability and poor HRQoL in our study. Interestingly, colchicine use was one of the determinants of functional disability and PCS. Colchicine is regarded as a useful drug to treat acute attacks of gout or to prevent attacks of gout [42]. However, side effects with this drug often include diarrhea, nausea, cramping abdominal pain and vomiting [43]. One explanation for this finding was that the existence of adverse effects encountered with colchicines leads to the decreased physical health and activities of daily living. Also consistent with past research, the use of glucocorticoids was associated with MCS [4]. Glucocorticoids are powerful drugs with strong anti-inflammatory and immu-nomodulatory effects that are often used to treat rheumatic diseases [44]. It should be noted that adverse effects (eg, skin thinning, cushingoid appearance) of glucocorticoids may be of great concern to patients [45]. Thus, the possible explanation may be that poor body image may influence the mental health of gout patients. Additionally, Scire and colleagues suggested that the relationship between corticosteroid use and mental component of the SF-36 is an issue that is still to be resolved [4]. Derijk et al. found that several of the mineralocorticorticoid receptor- and glucocorticoid receptor variants have been found associated with stress-related disorders, including depression, which have an effect on mental health [46]. Therefore, corticosteroids may impact HRQoL indirectly through psychological status. Further study could examine the impact of the inflammation of gout as a key likely factor in reduced HRQoL.

Current evidence suggested that psychological disorders were correlated with functional disability and HRQoL in the population with common chronic diseases [47–49]. A prospective cohort study from UK demonstrated that worse HRQoL was seen in gout people with anxiety and depression and depression was associated with functional disability [17]. As in our study sample, gout individuals with depression and anxiety had significantly lower scores in PCS and MCS. Meanwhile, the prevalence of depression and anxiety in the gout group with functional disability were higher than the gout group without functional disability. Notably, multiple regression analysis demonstrated that depression was significantly accounted for functional disability and PCS and anxiety was a predictive factor for MCS. From a psychological point of view, individuals with gout suffered from long-term pain, deformed joints, and frequent flares. They are concerned about loss of function, work disability and high costs of the disease. Hence, psychological care may be conducive to reducing functional disability and improving HRQoL of gout subjects. As we know, our study provided the first evidence of the effect of gout characteristics and anxiety on functional disability and HRQoL in gout individuals from China.

Though we reported predictors of functional disability and HRQoL in Chinese people with gout, we also had some limitations: (1) Functional disability, HRQoL, and psychological factors were measured by self-report questionnaires. (2) The sample size was recruited from a single clinic of rheumatology. Further longitudinal studies with objective functional disability, HRQoL, and psychological status measures from multi-centers should be conducted to develop the effective interventions to reduce functional disability and improve HRQoL of patients living with gout.

## Conclusions

In summary, this is the first known evaluation of contributors to functional disability and the effect of gout characteristics and anxiety on HQRoL in Chinese gout patients. The present study demonstrated that total pain, depression, and colchicine use were the most powerful predictors of functional disability in gout population from China. Cardiovascular disease, number of flares/last year, total pain, presence of tender joints, depression, anxiety, colchicines and corticosteroids use were independently risk factors for HRQoL. These findings emphasize the importance of psychological intervention and aggressive approach to relieve diseased-related symptoms and their psychological problems and finally to reduce functional disability and improve their HRQoL.

## Abbreviations

BMI: Body mass index
DM: Diabetes
GAD-7: Generalized anxiety disorder
HAQ-DI: Health assessment questionnaire-disability index
IQR: Interquartile range
MCS: Mental components summary
PCS: Physical components summary
PHQ-9: Patient health questionnaire
SD: Standard deviation
SF-36: the Short Form 36 Health Survey
sUA: Serum uric acid
VAS: Visual analog scale
WHR: Waist-hip ratio
